# Lithotomy

**Published:** 1889-10-26

**Authors:** 


### SURGERY.

### LITHOTOMY.

Mr. Vincent Jackson, F.R.C.S., publishes some statistics of perineal, lateral, and median lithotomy,* being operations performed on male infants and boys in the Wolverhampton and Staffordshire General Hospital from 1864 to 1888. During a period of twenty-four years 132 perineal operations for stone in the bladder of boys varying in age from one to fifteen years have been performed, five dying from causes sequential to the operation. Of the whole number the death ratio was one in a little over twenty-six cases. Although to a considerable extent Mr. Jackson is responsible for the performance of the operations, yet the series is not a personal one, as it comprises the operations of every member of the staff since 1864, and Mr. Jackson observes "it is a remarkable and almost unique record, and it nearly beats the barely five per cent. of deaths of Cheseden, who cut 213 patients and lost ten." Now, as a general rule, the operation for stone in the bladder in children is followed by favourable results, but after fifteen years of age a similar procedure is attended with progressively less favourable results as the age of the patient advances. Indian lithotomists have attained success equalling, if not excelling, those now given. A great deal depends upon whether the cases are selected or not. If the stone is not large, and if the patient is otherwise healthy, success after operation may be reasonably expected. But if the stone is large, and the patient scrofulous, or with large spleen from malarious causes, a favourable result is not so certain.

